# Correction: Optical properties and photoactivity of carbon nanodots synthesized from olive solid wastes at different carbonization temperatures

**DOI:** 10.1039/d2ra90127g

**Published:** 2022-12-16

**Authors:** Shadi Sawalha, Mohyeddin Assali, Ameerah Nasasrah, Maha Salman, Majd Nasasrah, Madleen Jitan, Hikmat S. Hilal, Ahed Zyoud

**Affiliations:** Department of Chemical Engineering, An-Najah National University Nablus Palestine sh.sawalha@najah.edu; Department of Pharmacy, Faculty of Medicine and Health Sciences, An Najah National University Nablus Palestine; Department of Chemistry, Faculty of Science, An-Najah National University Nablus Palestine

## Abstract

Correction for ‘Optical properties and photoactivity of carbon nanodots synthesized from olive solid wastes at different carbonization temperatures’ by Shadi Sawalha *et al.*, *RSC Adv.*, 2022, **12**, 4490–4500, https://doi.org/10.1039/D1RA09273A.

The authors regret that the name of one of the authors (Ahed Zyoud) was shown incorrectly in the original article. The corrected author list is as shown above.

The Royal Society of Chemistry apologises for these errors and any consequent inconvenience to authors and readers.

## Supplementary Material

